# Feather protein lysate optimization and feather meal formation using YNDH protease with keratinolytic activity afterward enzyme partial purification and characterization

**DOI:** 10.1038/s41598-021-93279-5

**Published:** 2021-07-15

**Authors:** Doaa A. Goda, Ahmad R. Bassiouny, Nihad M. Abdel Monem, Nadia A. Soliman, Yasser R. Abdel-Fattah

**Affiliations:** 1 Bioprocess Development Department, Genetic Engineering and Biotechnology Research Institute (GEBRI), City of Scientific Research and Technological Applications (SRTA-City), New Burg El-Arab City, Universities and Research Institutes Zone, Alexandria, 21934 Egypt; 2 Biochemistry Department, Faculty of Science, Alexandria University, Alexandria, Egypt

**Keywords:** Biotechnology, Microbiology

## Abstract

Incubation parameters used for the creation of a protein lysate from enzymatically degraded waste feathers using crude keratinase produced by the *Laceyella sacchari* strain YNDH were optimized using the Response Surface Methodology (RSM); amino acids quantification was also estimated. The optimization elevated the total protein to 2089.5 µg/ml through the application of the following optimal conditions: a time of 20.2 h, a feather concentration (conc.) of 3 g%, a keratinase activity of 24.5 U/100 ml, a pH of 10, and a cultivation temperature of 50 °C. The produced Feather Protein Lysate (FPL) was found to be enriched with essential and rare amino acids. Additionally, this YNDH enzyme group was partially purified, and some of its characteristics were studied. Crude enzymes were first concentrated with an Amicon Ultra 10-k centrifugal filter, and then concentrated proteins were applied to a "Q FF" strong anion column chromatography. The partially purified enzyme has an estimated molecular masses ranging from 6 to 10 kDa. The maximum enzyme activity was observed at 70 °C and for a pH of 10.4. Most characteristics of this protease/keratinase group were found to be nearly the same when the activity was measured with both casein and keratin-azure as substrates, suggesting that these three protein bands work together in order to degrade the keratin macromolecule. Interestingly, the keratinolytic activity of this group was not inhibited by ethylenediamine tetraacetic acid (EDTA), phenylmethanesulfonyl fluoride (PMSF), or iron-caused activation, indicating the presence of a mixed *serine*–*metallo* enzyme type.

## Introduction

In the poultry industry, feathers are a significant byproduct since they account for 5–7% of the body weight of the chicken. Feathers are constituted of 91% of keratin protein, 8% water, and 1% of lipid^1^. Several million tons of feathers are estimated to be created annually from the global poultry industry^2^.

The structural components of feathers have an elevated keratin content; furthermore, several tissues of reptiles, birds, amphibians, and mammals, such as hair, nails, horns, hooves, bones, fur, claws, hides, bird beaks, skin, wool, scales, and bristle, are made up of keratin^3^.

This protein source should be used in a large scale and be converted into value added product. Conventional methods of feather degradation, such as alkali hydrolysis and steam pressure cooking, not only destroy the amino acids but also require a huge amount of energy^4,5^. A cost-effective alternative way consists of biodegradation of the feathers by microorganisms using keratinase. Recently, many potential applications have been linked to keratinase and related products^6,7^, such as feather meal production, which is utilized as a supplement for feeding stuffs^7,8^. It is important to mention that the demand for industrial enzymes has progressively increased and is expected to rise at a compound annual growth rate (CAGR) of 5.8% between 2017 and 2022 and to reach USD 6.30 billion by 2022^9^.

The unique characteristic that distinguishes keratinase from other proteases is the ability to bind to complex and insoluble substrates (feathers, wool, silk, collagen, elastin, horns, hair, azokeratin, and nails)^9^. Although the mechanism of enzyme adsorption is not yet fully understood, it is known that the higher the adsorption capacity is, the higher the degree of keratin hydrolysis will be^10^.

Feather biodegradation by microbes producing keratinase or by direct enzymes formed by numerous microorganisms is considered to be an environmentally friendly approach to keratin waste recycling^7,8^. Therefore, this study explores a strategic cost-effective optimization protocol for the enzymatic production of FPL enriched with different amino acids from waste feathers using *Laceyella sacchari* YNDH keratinase. Additionally, feather meal formation was obtained as an end product of this biodegradation process. Finally, the intensive characterization of partially purified applied enzymes was explored.

## Results

### Optimization of the enzymatic degradation process of waste feather for Feather Protein Kysate (FPL) production and feather meal formation using crude YNDH protease/keratinase

In order to achieve the optimal response area for maximizing the protein contents of enzymatically degraded feathers in terms of protein conc. (µg/ml), significant independent variables [X1; time (h), X2; feather conc. (g), X3; keratinase activity (U/ml)] were further explored at three levels. Table 1 presents the design matrix of the variables in coded units with the experimental results of the protein lysate conc. All cultures were conducted in triplicate, and the obtained results were averaged.

**Table 1:** BBD for the three selected variables with coded and real values; observed results for the released protein concentration in the enzymatically treated feathers.

Trial	X1 Time (h)	X2 Feather conc. (w/v %)	X3 Keratinase (U)	X1 × X2	X2 × X3	X1 × X3	X1 × X1	X2 × X2	X3 × X3	Soluble protein conc. in FPL (µg/ml)
1	− 1(7)	− 1(1)	0(30)	1	0	0	1	1	0	797.50
2	1(21)	− 1(1)	0(30)	− 1	0	0	1	1	0	982.50
3	− 1(7)	1(3)	0(30)	− 1	0	0	1	1	0	1114.17
4	1(21)	1(3)	0(30)	1	0	0	1	1	0	1737.5
5	− 1(7)	0(2)	− 1(10)	0	0	1	1	0	1	194.16
6	1(21)	0(2)	− 1(10)	0	0	− 1	1	0	1	417.50
7	− 1(7)	0(2)	1(50)	0	0	− 1	1	0	1	402.50
8	1(21)	0(2)	1(50)	0	0	1	1	0	1	1649.1
9	0(14)	− 1(1)	− 1(10)	0	1	0	0	1	1	665.83
10	0(14)	1(3)	− 1(10)	0	− 1	0	0	1	1	1359.1
11	0(14)	− 1(1)	1(50)	0	− 1	0	0	1	1	849.16
12	0(14)	1(3)	1(50)	0	1	0	0	1	1	1774.1
13	0(14)	0(2)	0(30)	0	0	0	0	0	0	1619.1
14	0(14)	0(2)	0(30)	0	0	0	0	0	0	1670.8

*conc*. concentration.

### Multiple regression analysis and analysis of variance ANOVA test

**Figure 1:**
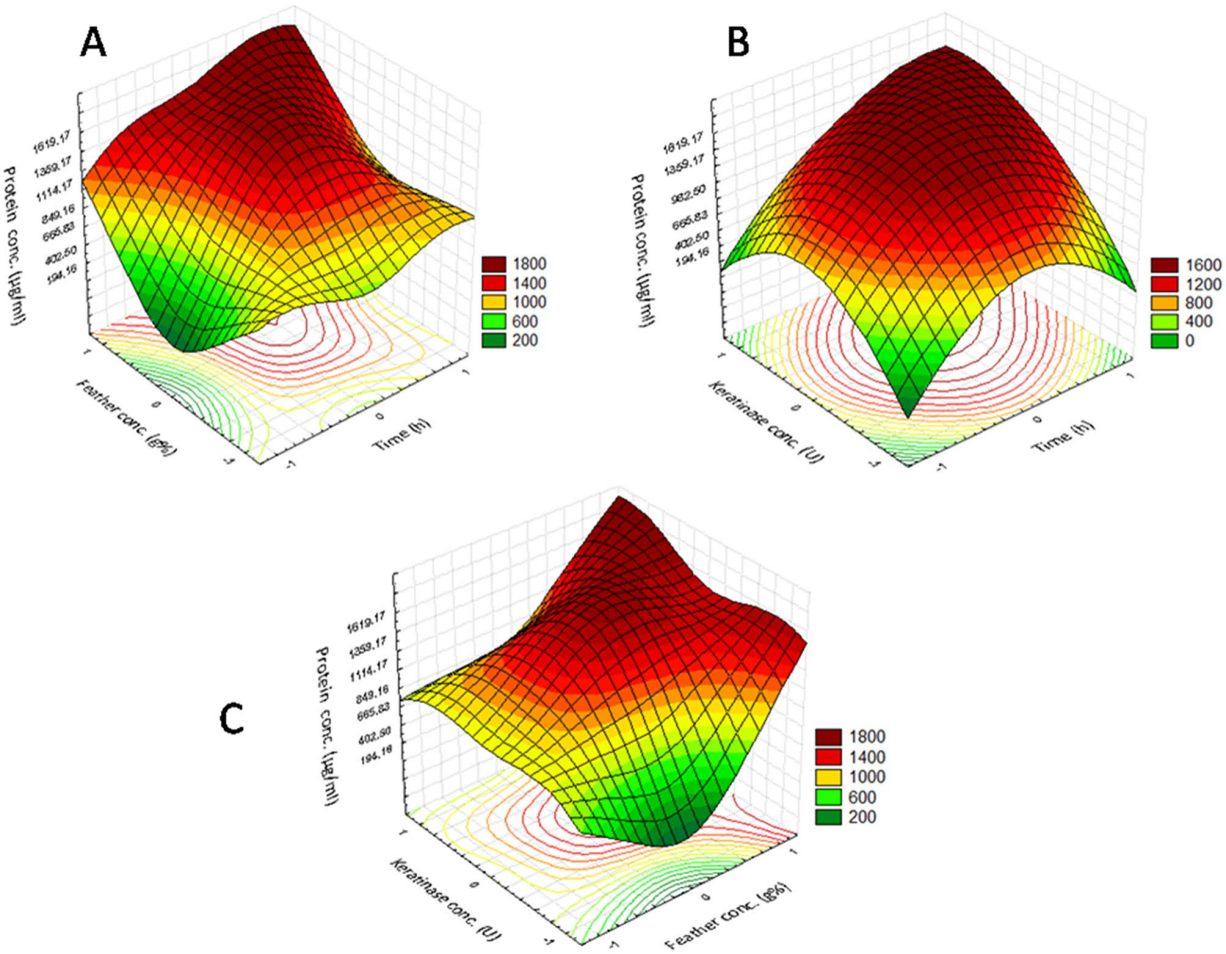
Three-dimensional surface plots showing the relationships between the significant variables and the protein concentration (µg/ml) in the FPL production; the optimal levels of the three factors were obtained from the maximum value of the polynomial model. (**A**) Interaction between the feather concentration and time; (**B**) interaction between the keratinase concentration and time; (**C**) interaction between the keratinase concentration and the feather concentration.

The three tested variables with fourteen trails were analyzed using the linear multiple regression analysis approach, and the corresponding percentage confidence rates were determined. Variance analysis was performed via ANOVA tests in BB experiments and is summarized in Table 2. The obtained result of *p* = 0.0235 indicates that there is a statistically significant relationship between the studied variables and the measured response, namely the protein conc. (µg/ ml). Additionally, the obtained value of the determination coefficient (*R*^2^ = 0.953), which is a measure of the fitting degree for the applied model, indicates that about 4.7% of the total variations in the measured response are not explained by the model. Additionally, the simultaneous effects of the three studied independent variables on the measured response are presented in Fig. 1 using three-dimensional graphs generated by the STATISTICA 5.0 software. The optimal values of the three studied variables obtained from the maximum polynomial model point were found to be 20.2 h, 3 g/100 ml, and 24.57 U/100 ml for the time, substrate conc., and enzyme conc., respectively, with the predicted calculated protein conc. being equal to 2159.85 µg/ml. Through the bench scale confirmatory test, a Y value of 2089.5 µg/ml was obtained, indicating that the calculated model accuracy was 96.7%. In this way, the conditions that were found to yield the highest conc. of protein lysate based on BB experiments were as follows: an incubation time of 20.2 h, a substrate conc. of 3 g%, an enzyme conc. of 24.57 U%, a pH of 10, and a cultivation temperature of 50 °C, with a measured protein content conc. of 2159.85 µg/ml.

**Table 2:** **S**tatistical analysis of BBD showing coefficient values, *t*-values, *p*-values, and confidence levels for each variable on the protein content of the enzymatically treated feathers alongside with the ANOVA results of the experiment.

Variables	Coefficients	Standard error	*t* Stat	*p*-value	Confidence level (%)
**Intercept**	1645	150.8	10.9	0.0004	
X1	284.7	75.40	3.77	0.0194	98.05
X2	336.2	75.40	4.459	0.0111	98.88
X3	254.7	75.40	3.378	0.0278	97.28
X1 × X2	109.5	106.6	1.027	0.362	63.77
X2 × X3	57.91	106.6	0.543	0.6159	38.40
X1 × X3	255.83	106.6	2.398	0.0744	92.55
X1 × X1	− 491.66	119.2	− 4.123	0.014	98.54
X2 × X2	4.583	119.2	0.0384	0.9711	2.882
X3 × X3	− 487.50	119.2	− 4.088	0.0149	98.50

ANOVA	Degree of freedom *df*	Sum of Squares *SS*	Mean Square *MS*	F Ratio *F*	*p*-value Significance *F*
Regression	9	3,770,892.116	418,988	9.209921	0.023528
Residual	4	181,972.4612	45,493.12		
Total	13	3,952,864.577			
Multiple R	0.9767				
R Square	0.9539				
Adjusted R Square	0.8503

### Amino acid analysis of feather meal

In this experiment, feather lysates were analyzed to find free amino acids released during feather degradation in both the cell-free supernatant after cultivation of *Laceyella sacchri* strain YNDH in the optimized medium and the feather hydrolysates produced due to an enzymatic treatment, as shown in Table 3, respectively. 16 amino acids were detected in both experiments and quantified as shown in Table 3. The presence of essential amino acids, such as leucine and isoleucine, indicates that the feather treatment performed with the native YNDH strain or the related crude keratinase enzyme provides feather lysate enriched with rare amino acids. The present study clearly shows that feather degradation by keratinase provided by the *Laceyella sacchari* YNDH strain is not only cost-effective but also a viable method to efficiently utilize disregarded feather waste. Additionally, the concentrations (concs.) of all the different types of amino acids produced through feather treatment using keratinase developed by *Laceyella sacchari* YNDH was found to be significantly higher than that in feather hydrolysate produced by applying the native organisms directly.

**Table 3:** Free amino acids and their concentrations in the cell-free supernatant.

Amino acid	Amino acid conc (μg/ml)	
	After growing the YNDH strain in the optimized medium containing 1% feather waste	After addition of the crude keratinase to the feather waste
Aspartic acid	56.852	587.76
Threonine	UD	164.74
Serine	32.58	145.32
Glutamic acid	42.227	577.44
Proline	3.067	887.76
Glycine	23.466	527.24
Alanine	6.724	686.06
Valine	6.992	339.3
Methionine	1.458	40.86
Isoleucine	3.124	199
Leucine	49.999	441.52
Tyrosine	7.555	348.9
Phenylalanine	18.866	749.9
Histidine	28.358	103.72
Lysine	6.413	213.68
Arginine	13.714	287.74

*UD* undetermined.

### Separation and collection of feather meal and protein lysate

**Figure 2:**
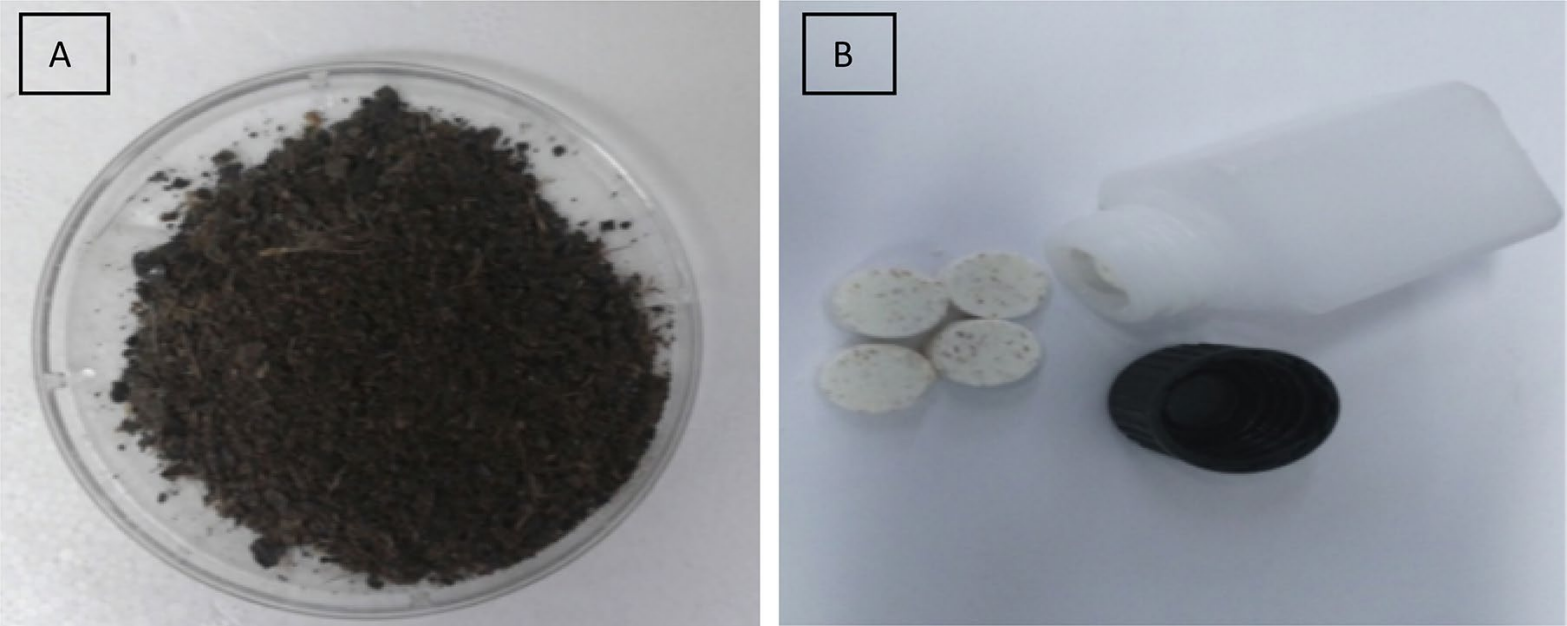
Final products: (**A**) Feather meal and (**B**) FPL consolidate.

**Figure 3:**
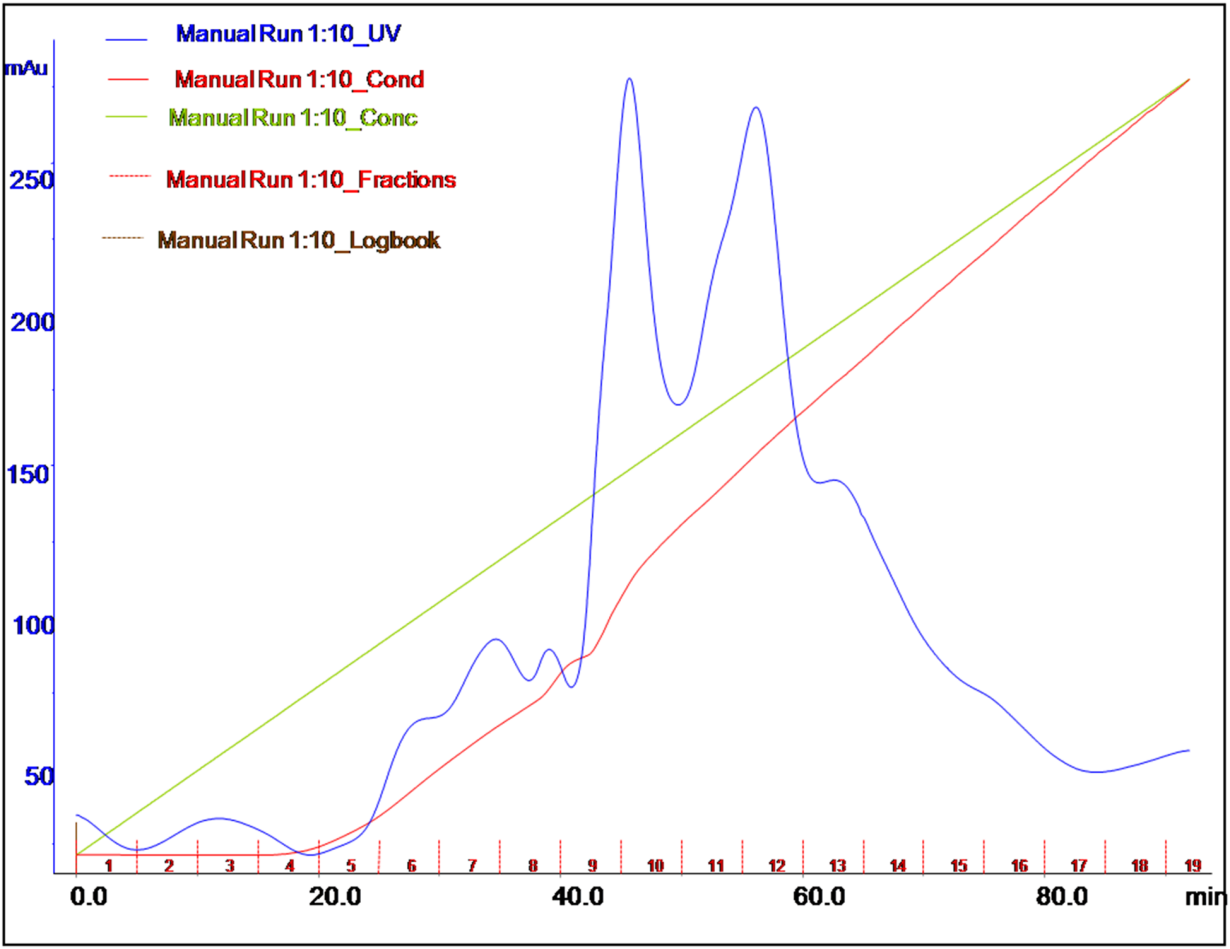
Elution profile of the protease/keratinase enzyme after loading on a Q FF strong anion exchange column.

After optimization of enzymatic degradation of waste feather by the YNDH protease/keratinase crude enzyme via the RSM in combination with Box-Behnken Design (BBD); the feather meal was settled down, centrifuged, and dried at 50 °C, at which point the supernatant-containing protein lysate was lyophilized, and the powder was consolidated, as shown in Figs. 2 and 3.

### Protease/keratinase purification

The cell-free supernatant developed after cultivation was collected and added to an Amicon Ultra centrifugal filter (with a cut-off of 20 kDa) to reduce the volume and remove protein impurities. This step is considered to be a partial purification step, as it increased the enzyme specific activity from 166.28 to 296.38 U/mg and the purification fold by a factor of 1.78, as shown in Table 4.

**Table 4:** Purification scheme of the protease/keratinase enzyme produced by *Laceyella sacchari* strain YNDH.

Purification steps	Total activity (U)	Total protein (mg)	Specific activity (U/mg)	Purification (fold)	Yield (%)
Crude (cell-free supernatant)	39,364	236.8	166.28	1	100
Concentrated crude (using Amicon Ultra 20 kDa)	16,953	57.2	296.38	1.78	43.06
Anion exchange using "QFF" strong anion column	5661	3.09	1881.3	6.34	14.38

**Figure 4:**
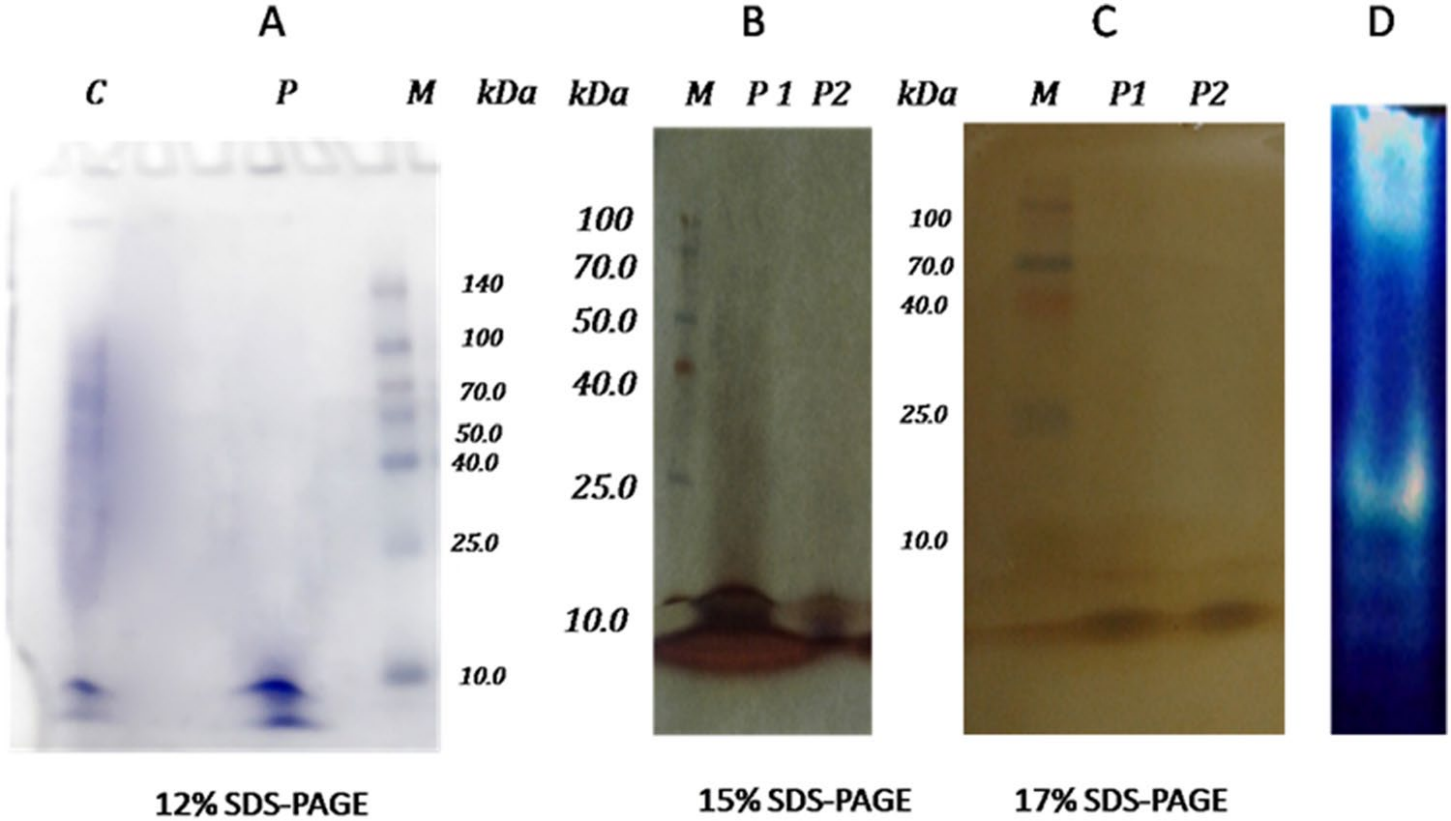
SDS-PAGE of the partially purified protease with keratinolytic activity from *Laceyella sacchari* YNDH. (**A**) SDS-PAGE (12%), cell-free supernatant (lane C), collected protease active fractions resulted from the anion exchange column (lane P), standard molecular mass marker (in the range of 10–140 kDa) (lane M), (**B**) SDS-PAGE (15%), standard molecular mass marker (in the range of 10–100 kDa) (lane M), collected protease active fractions resulted from the anion exchange column (lane p1, p2 repeats), (**C**) SDS-PAGE (17%), standard molecular mass marker (in the range of 10–100 kDa) (lane M), collected protease active fractions resulted from the anion exchange column (lane p1, p2 repeats), (**D**) Activity staining (zymogram) for a partially purified protease with keratinolytic activity from *Laceyella sacchari* YNDH (lane D).

For ion exchange chromatography, the working pH should be at least higher by one than the Isoelectric Point (IP) of the protein. At this pH value, the protein will possess a net charge sufficiently high to bind well to the ion exchange resin. The concentrated and desalted cell-free supernatant was subjected to a Q FF strong anion column chromatography, which was equilibrated with a 20-mM Tris base buffer with a pH of 8.5. The elution was performed with a 1 M NaCl solution in the same buffer. The elution profile (Fig. 3) reveals the presence of protease with keratinolytic activity in fractions of 6–8. Upon using the Q FF strong anion column, the enzyme was purified to an 11.31-fold with a specific activity and recovery of 1881.3 U/mg and 14.38%, respectively (Table 4). The pooled fractions were loaded and run on 12%, 15%, and 17% sodium dodecyl sulfate–polyacrylamide gel electrophoresis (SDS-PAGE) to determine the purity. Figure 4 illustrates the pattern of the protein profile before and after purification; the reduction in the number of protein bands to three at a molecular weight of 6–10 kDa can be noticed.

### Characterization of the partially purified protease/keratinase enzyme

Certain characteristics of the partially purified enzyme under study were evaluated using casein and keratin-azure substrates. Several parameters were investigated, including the optimal temperature, optimal pH, thermal and pH stability, some enzyme inhibitors/activators, surfactants, detergents, metal ions, and substrate conc.

### Optimal temperature and pH

**Figure 5:**
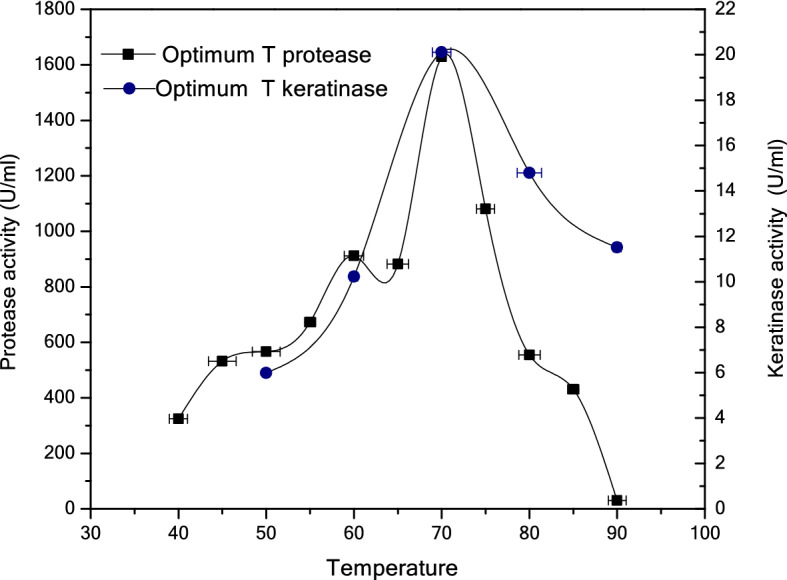
Optimal temperature for protease using casein as the substrate and for keratinase using keratin-azure as the substrate.

The effect of temperature on the partially purified enzyme was studied by measuring the activity at different temperatures ranging from 40 to 90 °C with a step of 5 °C and from 50 to 90 °C with a step of 10 °C using casein and keratin-azure as the substrate, respectively. The results presented in Fig. 5 show that the optimal temperature of the partially purified enzyme in terms of both the protease and keratinase activity was 70 °C; therefore, the enzyme can be classified as a thermoactive protease with keratinolytic activity, and the three isolated bands can work together as a keratinase group.

**Figure 6:**
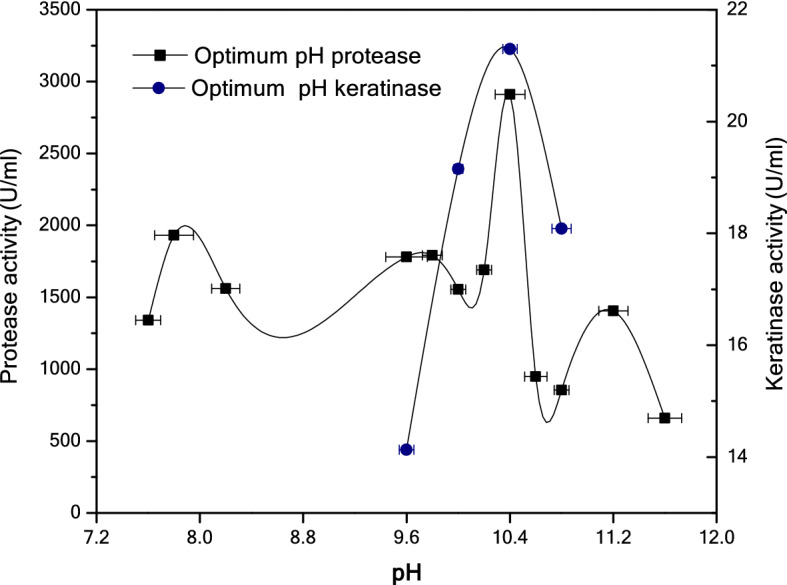
Optimal pH for protease using casein as the substrate and for keratinase using keratin-azure as the substrate.

By contrast, the effect of pH on the partially purified enzyme activity was investigated by determining the enzyme activity, firstly as protease for pH values of 7.6–11.6 and secondly as keratinase for pH values of 9.6–10.8. The pH activity profile of the enzyme (protease/keratinase) indicates that it exhibits a broad pH range with an optimal pH of 10.4, as shown in Fig. 6.

### Thermal and pH stability

In the present experiment, the effect of temperature on the thermal stability of the partially purified enzyme was studied by exposing it to a range of temperatures (50–55 °C) for 24 h and to specific temperatures (60 °C, 70 °C, and 75 °C) for 4 h; then, the residual activity was calculated considering that 100% is the enzyme activity at room temperature. The enzyme activity was measured as previously described and expressed in percent residual activity using casein/keratin-azure substrates (data not shown).

In summary, the results show that the enzyme was highly activated (238.46% as protease and 185.4% as keratinase) at a temperature of 50 °C; furthermore, it remained stable at 55 °C for up to 24 h. The enzyme retained its stability for 90 min at 60 °C; however, it lost about half of its activity at a temperature of 70 °C (the optimal temperature for enzyme activity) after only 60 min. Moreover, it was even less stable when exposed to higher temperatures (75 °C).

The pH stability of the partially purified enzyme was investigated as protease and keratinase by measuring the residual activity after incubating the enzyme at a pH ranging from 9.6 to 10.8 at room temperature for 60 min with time steps of 10 min and using casein and keratin-azure as substrates. The obtained results indicate that the enzyme was highly stable for all tested pH values (data not shown).

### Effect of certain metal ions, detergents, surfactants, solvents, and activators/inhibitors

The effect of certain metal ions on the partially purified enzyme activity was examined by measuring the residual activity in the presence of 1, 5, 10, 15, and 20 mM of each metal ion. All metal ions at concs. of 1, 5, and 10 mM were found to significantly activate the protease activity, whereas Hg^2+^ and Cu^2+^ inhibited the enzyme activity to 3.3% and 18.3%, respectively, when measured as protease. Moreover, the most significant metals capable to enhance the proteolytic activity were found to be Mg^2+^ and Fe^2+^:Mg^2+^ significantly increased the protease activity to 274.477% and 232.11% at 15 and 20 mM, respectively, whereas Fe^2+^ significantly enhanced the protease activity to 411.11% and 157.7% at 15 and 20 mM, respectively, as shown in Table 5. The response of the studied enzyme as keratinase to metal ions, detergents, surfactants, solvents, activators/inhibitors was also investigated at the most significant concs., namely 10 and 20 mM. As shown in Table 6, the keratinolytic activity of the enzyme was significantly enhanced (410.1% and 179.33%) by Fe^2+^ and Mg^2+^, respectively.

**Table 5:** Effect of different metal ions, detergents, surfactants, activators/inhibitors on the activity of the partially purified enzyme using the casein substrate and measured as protease.

Residual activity of protease % (Mean ± SD)					
Tested	Different metal ions at conc				
	1 mM	5 mM	10 mM	15 mM	20 mM
Cu^2+^	3.32 ± 0.50	4.66 ± 3.11	2.76 ± 1.13	2.33 ± 0.57	3.01 ± 0.33
Co^2+^	235.21 ± 6.31	378.61 ± 2.62	171.33 ± 2.91	151.50 ± 1.13	3.22 ± 0.01
Ca^+^	145.42 ± 3.01	323.79 ± 3.21	165.11 ± 6.22	136.10 ± 0.92	228.41 ± 2.52
Hg^2+^	18.37 ± 1.71	59.91 ± 1.82	16.00 ± 1.43	2.10 ± 0.15	2.72 ± 0.57
Mg^2+^	155.12 ± 0.99	281.31 ± 0.61	289.13 ± 3.95	274.40 ± 2.11	232.12 ± 1.73
Mn^2+^	157.81 ± 3.8	278.62 ± 0.92	98.44 ± 2.15	119.15 ± 2.32	1.23 ± 0.22
Fe^2+^	225.01 ± 1.34	195.71 ± 1.43	208.62 ± 1.63	411.10 ± 1.63	1577 ± 4.32
Ni^2+^	174.62 ± 4.02	260.22 ± 2.13	140.83 ± 1.84	134.12 ± 2.11	319.22 ± 0.05
Zn^2+^	244.51 ± 2.2	321.33 ± 1.24	92.66 ± 0.73	62.61 ± 1.92	0.92 ± 0.13
Control	100.00 ± 0.00	100.00 ± 0.00	100.00 ± 0.00	100.00 ± 0.00	100.0 ± 0.00

	Detergents, surfactants and solvents at conc				
	0.1%	0.5%	1%	2%	
Tween-20	114.62 ± 3.22	96.8 ± 1.80	127.6 ± 1.50	122.3 ± 1.1	
Tween-40	113.13 ± 2.7	177 ± 3.20	164.7 ± 1.52	149.3 ± 2.0	
Tween-80	96.24 ± 3.24	105 ± 0.90	122.8 ± 1.51	134.6 ± 1.4	
SDS	94.54 ± 0.41	21.3 ± 1.30	35.93 ± 1.00	0.7 ± 1.10	
Methanol	3.12 ± 1.04	7.0 ± 2.00	95.5 ± 0.94	100.8 ± 2.5	
Ethanol	7.45 ± 2.04	5.2 ± 2.70	125.2 ± 0.51	100.9 ± 1.6	
Glycerol	4.05 ± 1.03	6.6 ± 2.50	103.4 ± 1.5	104.2 ± 2.1	
Triton-X100	92.23 ± 1.32	195 ± 1.10	219.6 ± 1.60	4 ± 0.00	
Control	100.00 ± 0.00	100 ± 0.0	100 ± 0.00	100 ± 0.0	

	Activators/ inhibitors at conc				
	1 mM	5 mM	10 mM		
EDTA	94.31 ± 5.52	92.33 ± 1.03	93.52 ± 0.71		
DTT	102.11 ± 2.41	103.02 ± 2.05	100.23 ± 1.32		
PMSF	90.71 ± 2.12	112.92 ± 2.02	120.13 ± 0.40		
Control	100.00 ± 0.00	100.00 ± 0.00	100.00 ± 0.00

**Table 6:** Effect of the most promising concentration of metal ions, detergents, surfactants, activators, and inhibitors on the activity of the partially purified enzyme using the keratin-azure substrate and measured as keratinase.

Metals	Metal ions at conc. (20 mM)
Residual activity of keratinase % (mean ± SD)	
Cu^2+^	3.20 ± 2.96
Co^2+^	1.33 ± 2.30
Ca^+^	164.33 ± 4.50
Hg^2+^	4.66 ± 5.60
Mg^2+^	179.33 ± 5.10
Mn^2+^	3.66 ± 2.00
Fe^2+^	410.10 ± 4.50
Ni^2+^	120.30 ± 3.60
Zn^2+^	9.00 ± 4.00
Control	100.00 ± 0.00
**Detergents; surfactants and solvents at conc. (2%)**	
Tween-20	161.20 ± 0.23
Tween-40	205.40 ± 3.14
Tween-80	165.75 ± 2.40
SDS	62.45 ± 30
Methanol	92.00 ± 2.00
Ethanol	71.70 ± 2.00
Glycerol	136.40 ± 3.00
Triton-X100	85.00 ± 2.00
Control	100.00 ± 0.00
**Activators/inhibitors at conc. (10 mM)**	
EDTA	114.8 ± 3
DTT	124.7 ± 1.5
PMSF	176.07 ± 3.1
Control	100 ± 0.0

Furthermore, the enzyme activity was investigated as protease and keratinase activity in the presence of certain surfactants, detergents, and solvents. The results presented in Table 5 reveal a slight increase in the activity of the partially purified protease/keratinase enzyme when treated with Tween-20 and Tween-80 at different concs. In particular, Tween-40 was found to increase considerably the proteolytic and keratiolytic activity (to 149.3% and 205.4%, respectively). A sharp increase of 219.6% in the proteolytic activity was obtained by incubating the enzyme with Triton-X-100 at 1%; however, this did not result in any change in the keratinolytic activity. On the other hand, a sharp decrease in the proteolytic/keratinilytic activity was obtained by incubating the enzyme with SDS. Moreover, ethanol, methanol, and glycerol had no effect on the protease/keratinase enzyme activity, whereas glycerol was found to enhance the keratinase activity.

In addition, the enzyme activity was investigated in the presence of some activators and inhibitors, such as EDTA, an inhibitor of *metallo*-type proteases, dithiothreitol (DTT), a reducing agent, and phenylmethanesulfonyl fluoride (PMSF), a serine protease inhibitor. The results presented in Tables 5 and 6 show that both EDTA and DTT caused a slight increase in the activity of the partially purified protease/keratinase when measured using both casein and keratin-azure as substrates. By contrast, PMSF significantly increased the enzyme activity as both protease (121.13%) and keratinase (176.07%) at a conc. of 10 mM.

### Effect of the substrate conc.

**Figure 7:**
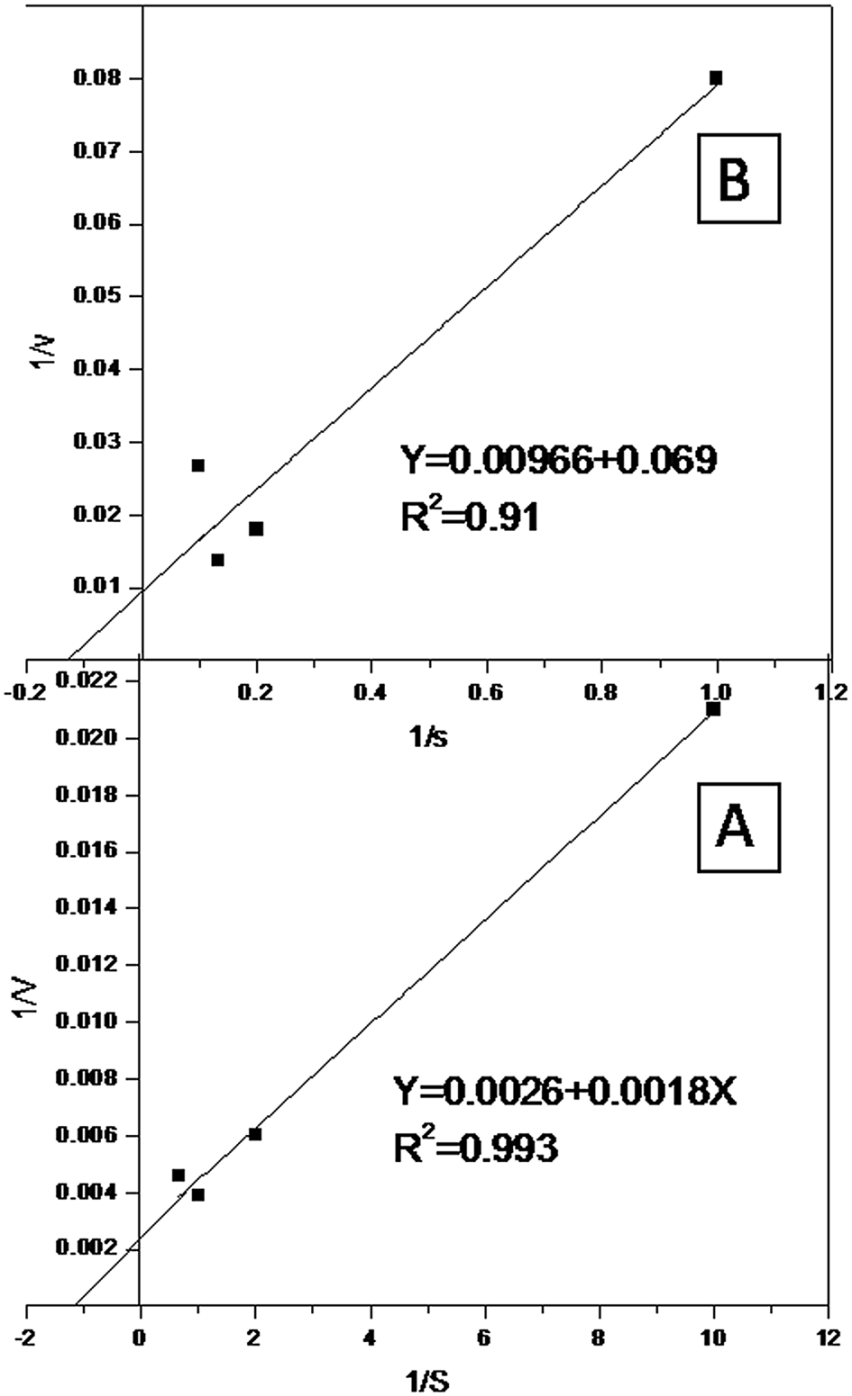
Lineweaver–Burk plot of the partially purified enzyme. (**A**) Protease using casein as the substrate and (**B**) keratinase using keratin-azure as the substrate.

The kinetic parameters (*K*_m_ and *V*_max_) of the extracellular YNDH protease/keratinase enzyme for hydrolysis of casein and keratin-azure were determined via the Lineweaver–Burk double-reciprocal plot at a temperature of 70 °C and for a pH of 10.4. The hydrolysis efficiency for casein, represented by *K*_m_ and *V*_max_, is shown in Fig. 7A; the values of *K*_m_ and *V*_max_ are 7 mg/ml and 384.6 U/mg, respectively. On the other hand, the hydrolysis efficiency for keratin azure is shown in Fig. 7B; in this case, the values of *K*_m_ and *V*_max_ are 7.2 mg/ml and 103 U/mg, respectively. The estimated *K*_m_ value indicates the affinity of the enzyme toward the substrate, whereas *V*_max_ indicates the catalytic activity of an enzyme and is usually desired to be as high as possible. The high *V*_max_ estimated indicates the high affinity of the tested enzyme toward the substrate.

## Discussion

Keratinases are very interesting from a biotechnological point of view; this is particularly true for keratinases of microbial origin, compared with plant or animal sources, since they possess almost all the features desired for biotechnological applications. Among these biocatalysts, high alkaline proteases, which alone account for about 40% of the global enzyme sales worldwide^11^, are particularly suitable for industrial use due their high stability and performance in tough conditions.

Feathers can be degraded to feather meal to use as animal feed, organic fertilizers, and feed supplements, as they consist of > 90% protein and are rich in hydrophobic amino acids and major amino acids, such as cysteine, arginine, and threonine^6,12^. Hydrothermal processes are the most common methods for obtaining feather meal; in these processes, feathers are cooked at a high temperature and pressure. However, hydrothermal treatment results in degradation of essential amino acids, such as methionine, lysine, tyrosine, and tryptophan. Additionally, feather meal resulted from hydrothermal treatment is usually has a poor digestibility and low nutritional benefits^4,13^*.*

In this respect, the microbial degradation of feathers into feather meal has become more significant; thus, new microbes are being considered for the efficient degradation of feathers. Bacterial pure keratinase are reported to degrade 10–20% of feathers in the absence of live or chemical redox, as reported by Hossain et al.^14^. On the other hand, feathers treated by keratinase from either *Bacillus licheniformis* ER-15 or *Bacillus paralicheniformis* MKU3 are mostly degraded (up to 90%), as has been reported by many research groups^15,16^. Similarly, the studied keratinase of the *Laceyella sacchari* YNDH strain was observed to completely degrade feathers into feather meal and protein lysate; in order to achieve this particular process, the parameters that regulate the enzymatic degradation of feathers into protein lysate were studied using the BBD. Further analysis of the studied variables (time, feather conc., and enzyme conc.) was performed at three levels, namely − 1, 0, and + 1, to address the optimal response region of the protein conc. in the feather lysate treated by the YNDH crude protease with keratinolytic activity. The *R* value for the protein conc. (μg/ml) was 0.97 in this experiment. This value indicates a high correlation between the experimental value to the predicted one. The optimal conditions obtained from the optimization experiment were verified experimentally and compared to the optimal value predicted by the model. The estimated protein conc. was 2089.5 µg/ml, whereas the predicted value from the polynomial model was 2159.8 µg/ml. This high degree of accuracy (96.7%) is an evidence of the model accuracy under the following optimal conditions: a time of 20.2 h, a feather conc. of 3 g%, a keratinase activity of 24.5 U/100 ml, a pH of 10, and a cultivation temperature of 50 °C. This result is in agreement with that obtained by Kumar et al.^17^. In this previous work, the authors reported that, for a feather concentration of 60 g/l, the protease, amino acid, peptide, and soluble protein production was reduced, and the maximum productivity of the soluble proteins and peptides was obtained in a culture medium containing 30 g/l of feathers.

The current process of bioconversion of feathers into FPL and feather meal is an enzymatic process in which no additional redox reactions are observed. The process is not only simple and time-saving, but is also economically viable as it does not require any bioreactor for feather degradation. Thus, bulk feathers can be easily recycled into amino acids and feather meal using this predicted newly found keratinase produced by the *Laceyella sacchari* strain YNDH within 20 h.

Worldwide, commercial poultry processing generates more than a million tons of feathers per year^18^, which are currently converted to feather meal via steam pressure and chemical treatments. However, these methods degrade amino acids and require significant energy input. Consequently, keratin can be transformed into usable biomass, protein concentrates, or amino acids using keratinolytic microorganism-derived proteases^8^. The nutritional improvement of feather meal and the use of microbial FPL in feed studies have shown that keratinase therapy may significantly increase the digestibility of amino acids in feather keratin^19,20^.

In the present study, the analysis of feather lysate obtained from the effect of the crude keratinolytic protease of *Laceyella sacchari* YNDH showed the presence of 16 different amino acids. Feather hydrolysate contained a good amount of essential amino acids (phenylalanine, valine, leucine, and isoleucine) and rare amino acids, such as serine and proline, along with other amino acids, particularly the very important sulfur-containing amino acids, such as methionine. These findings are in agreement with the results reported by Ganesan and Rajarajan^21^, namely that the feather lysates developed by a thermotolerant *Streptomyces graminofaciens* contained 20 amino acids. Additionally, in the present study, it was observed that the conc. of all different types of amino acids in FPL (i.e., feathers treated by the enzyme) was significantly higher than that in feather hydrolysate produced by the native organisms directly. These findings are in agreement with those reported by Tiwary^5^, who found that, during fermentation, more than 0.5–2% w/v of feathers can be used, and essential amino acids are utilized by microorganisms that decrease the nutritional value of feather meal.

To further understanding the applied YNDH enzyme, which is characterized by the dual protease/keratina function, purification and intensive biochemical characterization were conducted. The purity was gradually improved through successive purification steps, from the crude extract to the final separation step using anion exchange (i.e., the Q FF strong anion column). This revealed three distinctive bands with low molecular weights (≤ 10 kDa) indicating a partial purification. Generally, the molecular weights of bacterial keratinases are different among different bacterial species. However, the results obtained in the present study are in contrast with those reported by other studies^22,23^. Indeed, keratinase with a relatively low molecular mass (18 kDa) was observed in *Streptomyces albidoflavus*, whereas a higher molecular weight was observed in *Kocuria rosea* (240 kDa), in agreement with the results reported by Lange^24^, which revealed that a combination of endoprotease, exoproteases, and oligopeptidase is required to promote keratin degradation. Hence, this work shows a novel group of keratinases with a very low molecular weight equal to or below 10 kDa.

The effect of temperature on the activity of the partially purified protease with keratinolytic activity indicates that isolated enzymes and/or isozymes are capable of hydrolyzing casein and keratin-azure substrates in a wide range of temperature from 40 to 75 °C; however, the hydrolysis maximum temperature when using both casein and keratin-azure substrates is 70 °C. Therefore, this protein could be classified as a thermoactive protease/keratinase enzyme, and the three isolated bands can work together as a keratinase group. Similarly, Habbeche^25^ reported that the optimal temperature for keratinase is 70 °C. On the other hand, the pH activity profile of this enzyme indicates a broad pH range (7.6–11.6) of activity, with a maximum at a pH value of 10.4 when using casein/keratin-azure substrates. In summary, the optimal protease/keratinase activity was observed at a pH of 10.4; however, by increasing the pH above 10.4, a rapid decline in the protease activity was noticed. These findings are in agreement with several earlier reports showing that the optimal pH value of alkaline protease is close to 10^26–29^.

The thermal stability of the partially purified protease/keratinase indicates that it was activated at 50 °C for 24 h. Additionally, it retained its stability for 90 min at 60 °C. On the other hand, the investigated enzyme was completely inactive at 75 °C after only 20 min. The increased proteolysis rate of proteases at elevated temperatures has been reported to be one of the factors responsible for the rapid thermal inactivation of these enzymes, as described by Ghorbel et al.^30^. This is in agreement with the alkaline protease obtained from *Bacillus subtilis*^26,31^ and from *Streptomyces clavligerus*^32^, where the enzyme was stable at a pH of 9.6–10.8 after a 1-h incubation period at each tested pH value. The effect of different metal ions and chemical compounds on the activity of the partially purified protease/keratinase enzyme indicates that the enzyme activity was completely inhibited by 1 mM HgCl_2_ and CuSO_4_; these findings are in agreement with those obtained by Patel and Sana et al.^28,33^. By contrast, the Fe^2+^ metal ions caused an improvement in the activity. This indicates that these ions have a functional role in the molecular structure of the enzyme, meaning that the studied enzyme is related to a *metallo*-type enzyme. It is unlikely that the iron metal caused a decreasing effect on alkaline protease produced by *Bacillus* sp. and *Bacillus aquimaris* VITP4 as reported by some researchers^34,35^. Additionally, most of the microbial *metallo*-proteases are Zinc dependent^36^.

On the other hand, non-ionic surfactants, such as Tween-20, Tween-40, Tween-80, and Triton-X-100, induce a slight enhancement of the enzyme activity upon increasing the conc. of the tested surfactants from 0.1 to 2% or Triton from 0.1 to 1%, which is in agreement with the findings reported by Joo et al. and Sana et al.^31,33^. They reported an enhancement in the alkaline protease activity with the use of Triton-X-100 and Tween-80. Additionally, PMSF is known to sulphonate the essential serine residue in the active site of the protease, resulting in a total loss of the enzyme activity^37^. It is unlikely that PMSF caused the activation of the studied partially purified protease/keratinase at a conc. of 10 mM. Keratin is known as a complicated macromolecule, it needs extra and intra-enzymes to be reach the complete degradation as described by Singh and Kushwaha^38^. Keratinase is considered a broad spectrum protease with the unique ability to degrade keratin as reported by Wan-Ling et al.^39^. Additionally, keratinases of microbial origin are grouped into *metallo*, *serine*, or *metallo–serine* as stated by Brandelli^40^. Accordingly, the studied enzyme could be classified as a mixed-group *metallo–serine*, as it is activated by iron and not inhibited by either EDTA or PMSF. This mixture group is working in a synergistic way in order to hydrolyze the insoluble macromolecule of keratin.

Furthermore, the present work shows that the studied keratinase enzyme group has the same affinity toward keratin-azure and casein since the *K*_m_ value for keratin azure is nearly the same as that for casein. It is known that *K*_m_ is independent of the enzyme conc. and is rather an intrinsic characteristic of an enzyme at specific temperature and pH values^41^. Higher *V*_*max*_ and lower *K*_*m*_ values indicate a higher efficiency of protease with keratinolytic activity from *Laceyella sacchari* YNDH.

The present study presents the first report of enzymatic degradation of waste feather for FPL production and feather meal formation using a newly found crude protease/keratinase from a local isolate of *Laceyella sacchari* YNDH. In addition, the characteristic features of the partially purified enzyme concerning its unusually low molecular weight renders it possible to classify this enzyme as a novel protein.

## Conclusions

In this work, it was shown that the protease group with keratinolytic activity produced by the *Laceyella sacchari* strain YNDH may be a novel protein due to its unusual features. The enzyme was purified using Fast Protein Liquid Chromatography (FPLC) to a purification fold of almost 11.31 and specific activity of 1881.3 U/mg. The molecular weight of the partially purified enzyme using the SDS-PAGE technique was found to be lower than 10 kDa, representing three active subunits. The enzyme possesses contradicting features, justifying its classification as a *serine–metallo* keratinase, which is an excellent enzyme required for the degradation of keratin macromolecules. Furthermore, it was demonstrated that the optimization of the enzymatic feather-lysate production is enriched with amino acids by the crude enzyme syrup through the RSM approach. The optimal conditions required for promoting the FPL were as follows: an incubation time of 20.2 h, a substrate conc. of 3 g%, an enzyme conc. of 24.57 U%, a pH of 10, and a cultivation temperature of 50 °C, with a measured protein conc. of 159.85 µg/ml.

## Materials and methods

### Enzyme production and preparation

The protease/keratinase enzyme was produced in the optimized medium, as previously reported by Goda et al.^42^. After a 48-h fermentation time, broth was collected in a beaker and kept at room temperature in static conditions for 2 h. Most of the bacterial cells were placed with the feathers at the bottom of the beaker; the supernatant was decanted and then filtered through 0.2-μ filters (MDI, India) using a vacuum pump (WATSON-MARLOW-101 U/R). This micro filtered supernatant was concentrated and used as the crude enzyme through the lyophilization technique, in which the produced powder was dissolved in a 0.1 M glycine–NaOH buffer with a pH of 10 and stored at 4 °C. The concentrated enzyme was used for feather degradation and feather meal production after obtaining the required dilution through direct addition to the feather waste.

### Feather procurement

Chicken feathers were procured from the local market; they were washed with detergent followed by distilled water. The washed feathers were then dried for 8 h at 50 °C and used for the subsequent experiments.

### Optimization process via RSM (BBD)

BB experiments were implemented in the present work to maximize the nutritional content (mainly consisting of soluble protein) of feather-lysate during the feather meal creation process. Cleaned dried feathers were used as a biological substrate for the applied YNDH protease/keratinase crude enzyme, where the waste was processed in a glycine–NaOH buffer with a pH of 10 and incubated with the enzyme at 50 °C.

The RSM was used to optimize the protein content of the feather-lysate generated due to the enzyme. An enhancement of the protein conc. of lysate is considered to be the main aim of the BBD. Three variables (incubation time, substrate concentration, and enzyme concentration) were studied at three levels (high, medium, and low), which were indicated by the levels + 1, 0 and − 1, respectively, as shown in Table 1^43^.

As shown in Table 1, a design matrix consisting of 14 trials, was created to study the interaction between significant variables affecting feather degradation by the crude YNDH protease/keratinase enzyme; in these trials, the soluble protein concentration was considered to be the main response (μg/ml). Aliquots were collected at specific times (7, 14, and 21 h) and centrifuged for 10 min at 10,000 rpm; then, the protein content of the clear supernatant was measured at 750 nm (μg/ml) after subtracting the blank, which was taken at time zero. All cultures were conducted in triplicate, and the obtained results were averaged. This optimization method includes three key steps: conducting the statistically designed experiments, estimating the standardized mathematical model coefficients, and predicting the model answer and testing its adequacy^44^.

### Enzyme activity

Protease activity was measured using casein as the substrate. A cell-free supernatant was used as the source of the enzyme, where 0.25 ml of an appropriately diluted solution was mixed with 0.25 ml of 1% casein prepared in a 100-mM glycine–NaOH buffer (with a pH of 10) and then incubated for 10 min at 60 °C. The reaction was stopped by adding 0.750 ml of 5% trichloroacetic acid (TCA). The mixture was then centrifuged at 13,000 rpm for 10 min to remove the precipitate. The absorbance of the soluble fraction was estimated at 280 nm against the blank, and a standard curve was generated using l-Tyrosine^45^. By contrast, keratinolytic activity was measured using keratin-azure (Sigma-Aldrich, USA) as the substrate; it was first frozen at − 20 °C and then grinded into a fine powder. 5 mg/ml of keratin-azure powder was suspended in a 100-mM glycine–NaOH buffer (with a pH of 10). The reaction mixture contained 1 ml of the enzyme solution and 1 ml of the keratin-azure suspension. The reaction was carried out at 60 °C in a water bath with an agitation rate of 200 rpm for 30 min. The mixture was boiled for 5 min after the incubation period and centrifuged at 5000 rpm for 20 min to remove the substrate. The supernatant was spectrophotometrically measured at 595 nm for the release of azo dyes. A mixture of the enzyme and substrate was boiled before being used as a control sample. All assays were performed in triplex reactions^46^.

### Total protein estimation

The Lowry method was applied to estimate the total soluble protein conc. using the standard curve of bovine serum albumin^47^.

### Amino acid analysis

An amino acid analyzer for chromatography columns and the consumable catalogue (SYKAM) was used to evaluate the amino acid content of soluble protein lysate; the process is based on a high-performance liquid chromatography technique. Sample hydrolysates were prepared following the method proposed by by Moore and Stein^48^. A hydrolysis tube was filled with 200 mg of the sample. The sample was then attached to a tube containing 5 ml of 6 M HCl, which was tightly closed and incubated at 110 °C for 24 h. The solution was filtered after incubation, and 200 ml of the filtrate was evaporated to dryness at 140 °C for 1 h. Each hydrolysate after dryness was diluted with 1 ml of 0.12 M citrate buffers (with a pH of 2.2), and the same standard was applied to amino acids. At 130 °C, a 150 μl aliquot of the sample hydrolysate was injected into a cation separation column. At a flow rate of 0.7 ml/min, a ninhydrine solution and an eluent buffer (the buffer system included sodium acetate (90%) and acetonitrile (10%) were delivered simultaneously into a high temperature reactor coil (16 m in length). To speed up the chemical reaction of the amino acids with ninhydrine, the buffer/ninhydrine mixture was heated in the reactor at 130 °C for 2 min. A dual channel photometer was used to detect the reaction mixture's products at wavelengths of 570 and 440 nm. The amino acid composition was determined using the integrator's areas of the standards and expressed as a percentage of the total protein amount in µg/ml.

### Consolidation technique

In order to use feather-lysate enriched with a soluble protein as an animal supplement in an adequate form, feather-lysate was lyophilized using a lyophilizer (Acculab, USA) to concentrate it and was compressed into a dense solid using the cold pressing (CP) technique (hydraulic pressing machine used for cold pressing consolidation). For cold pressing of protein lysate, a simple hydraulic press with a 10-mm pressing die was used. The protein lysate content in powder form was placed in a die, normally made of stainless steel, under a hydrostatic pressure of 5 t (i.e., 0.6 GPa), and this pressure was sustained for 6 min, as shown in Fig. 2.

### Anion-exchange chromatography for protein purification

The concentrated enzyme solution (10 ml) was placed into an anion exchange (Q FF strong column) coupled with a 20-mM Tris base buffer with a pH of 8.5 using an automated ÄKTAprime plus method for the purification of protease/keratinase. The column was washed with five volumes of balancing buffer columns to remove the unbound proteins. The column-bound proteins were eluted with a linear gradient (0–100 M) of NaCl in a 20-mM Tris base buffer with a pH of 8.5 at a flow rate of 1 ml/min and fraction size of 5 ml. Fractions containing keratinolytic protease were pooled, desalinated, and concentrated using the Amicon-10 ultrafiltration concentrator (with a 20-kDa cut-off membrane).

### SDS-PAGE and zymography

SDS-PAGE was used to test the protein purity and to detect the protein molecular mass as defined by Laemmli^49^ at polyacrylamide concs. of 12%, 15%, and 17%. In these measurements, a protein standard was used, and the same conditions as those used by Laemmli^49^ were adopted. After running, the gel was stained with Coommassie Brilliant Blue R-250 with an SDS of 12% and with silver with an SDS of 15% and 17%^50^. Zymography is a simple and sensitive electrophoretic approach that was used by Leber^51^ to image the protein bands of protease with keratinolytic activity as transparent bands against a dark background, following a simple renaturation and staining procedure.

### Enzyme characterization

The partially purified enzyme was characterized by studying the effects of temperature and pH on the activity and stability of the enzyme. Additionally, the effects of certain metal ions, surfactants, inhibitors, and detergents were tested. The effects of different substrate concs. (casein/keratin-azure) were also investigated.

### Effect of temperature and pH

To study the effect of temperature on the protease/keratinase enzyme activity, the reaction mixture was incubated at different temperatures ranging from 40 to 90 °C and from 50 to 90 °C in a 100-mM glycine–NaOH buffer with a pH of 10, using casein and keratin-azure as substrates, respectively. The enzyme activity at different temperatures was determined and expressed in percentage relative to the maximum value (100%). By contrast, to study the effect of pH on the activity, the reaction mixture (enzyme with casein as the substrate) was incubated at different pH values (7.6–11.6). The substrate was dissolved individually in 100 mM of buffers: tris–HCl (pH of 7.6–8.2), glycine–NaOH (pH of 9.6–10), NaHCO_3_–NaOH (pH of 10–10.8), and Na_2_HPO_4_–NaOH (pH of 11.2–11.6). The enzyme activity at different pH values was determined and expressed in percentage values as the relative activity, and the reaction mixture was incubated at the optimal temperature for 10 min. When keratin-azure was used as the substrate, it was dissolved individually in 100-mM buffers: glycine–NaOH (pH of 9.6–10) and NaHCO_3_–NaOH (pH of 10–10.8). The enzyme activity for the tested alkaline pH values was determined and expressed in terms of the percentage relative activity, and the reaction mixture was incubated at the optimal temperature for 30 min.

### Thermal and pH stability

The thermal stability was evaluated by incubating the partially purified enzyme at a temperature in the range of 50–70 °C. The enzyme was preincubated at temperatures of 50 °C and 55 °C, and the samples were withdrawn every hour over a 24-h period; by contrast, at temperatures from 60 to 75 °C, the samples were withdrawn every 10 min over a period of 240 min. The enzyme activity was measured after exposure to these conditions using casein and keratin-azure as substrates and was expressed in terms of the percent residual activity. To study the pH stability, the enzyme was preincubated for 1 h at room temperature at different pH values (9.6–10) and (10–10.8) using glycine–NaOH and NaHCO_3_–NaOH, respectively. Aliquots of the mixtures were taken every 10 min to measure the residual enzyme activity (%) in the control sample (untreated enzyme), where the optimal conditions of temperature and pH were used in the reaction mixtures. The pH stability was tested for all samples using casein and keratin-azure as substrates.

### Effect of certain metal ions, surfactants, solvents, activators, and inhibitors

The effect of cetrain metal ions on the enzyme activity of the partially purified enzyme was investigated using CaCl_2_, MgSO_4_, ZnSO_4_, HgCl_2_, CuSO_4_, KCl, MnCl_2_, FeSO_4_, NiSO_4_, CoCl_2_, and FeSO_4_ at final concs. of 1, 5, 10, 15, and 20 mM. The enzyme was preincubated with the tested metal ion for 15 min at room temperature. The effects of some surfactants, such as Triton X-100, Tween 20, 40, 80, and SDS, on the enzyme activity were tested at final concs of 0.1%, 0.5%, 1%, and 2%. Furthermore, the influence of several solvents, including ethanol, methanol, and glycerol, on the protease activity was tested at final concs of 1% and 2%. In addition, the influence of some inhibitors, such as PMSF, DTT, and EDTA, on the enzyme activity was investigated at final concs. of 1, 5, and 10 mM. The enzyme was preincubated with each chemical (either solvent or inhibitor) for 15 min at room temperature; then, the residual activity was determined, as mentioned before, using casein as the substrate for all concs., and the more promising concs. (final conc.) were tested using keratin-azure as the substrate. The enzyme activity without any metal ion or agent was as assumed to be 100% under optimal conditions of temperature and pH (Supplementary Information).

### Effect of different substrate concs.

The kinetic constants (*V*_*max*_ and *K*_*m*_) were determined using the Lineweaver–Burk double-reciprocal (1/*v* versus 1/*S*) plot^52^ by evaluating a fixed amount of the enzyme as a function of different final concs of the casein substrate (0.1–2%) prepared in a 100-mM NaHCO_3_–NaOH buffer with a pH of 10.4, which was incubated for 10 min at 70 °C. Additionally, in the case of the keratin-azure as the substrate, the kinetic constants were determined by evaluating a fixed amount of the enzyme as a function of different final concs of the keratin-azure substrate (1–10 mg) prepared in a 100-mM NaHCO_3_–NaOH buffer with a pH of 10.4, which was incubated for 30 min at 70 °C.

## Data Availability

All data produced during this study are included in this published article.
